# Supplementary material for: A Global Screen for Assembly State Changes of the Mitotic Proteome by SEC-SWATH-MS
Source: Cell Syst. 2020 Feb 26;10(2):133–155.e6. doi: 10.1016/j.cels.2020.01.001 (PMC7042714; doi:10.1016/j.cels.2020.01.001)

Q15011 | HERP1_HUMAN | HERPUD1 HERP KIAA0025 MIF1
Monomer MW [kDa]: 43.72 Monomer expected elution fraction: 47

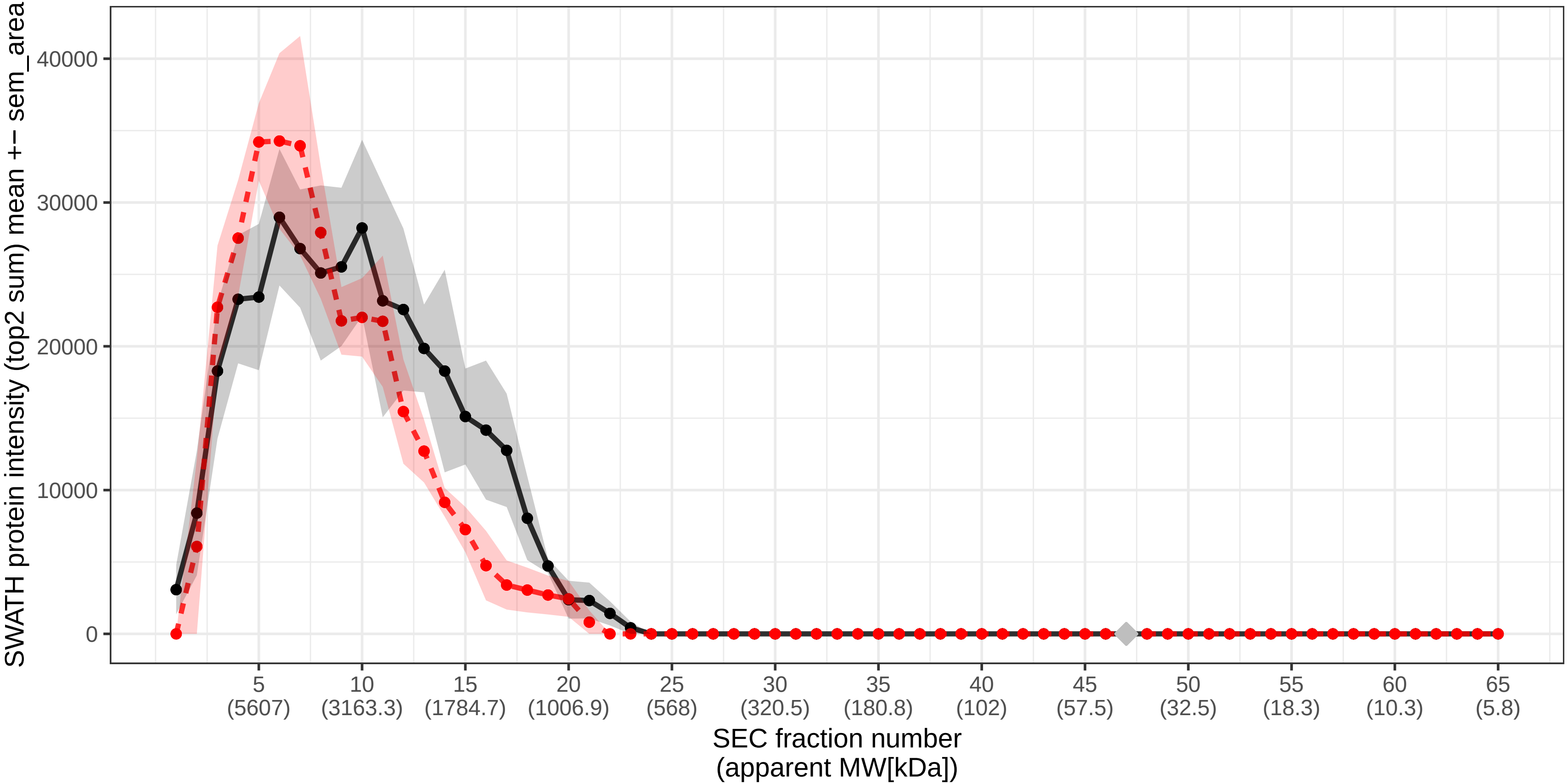

Supplement: Data S1. SEC-SWATH-MS Protein Chromatograms, Related to Figure 1 [file mmc6.zip › SECchrom_Q15011_HERP1_HUMAN_HERPUD1_HERP_KIAA0025_MIF1.pdf]
